# Case report: Cytopenias in VEXAS syndrome - a WHO 2022 based approach in a single-center cohort

**DOI:** 10.3389/fimmu.2024.1354130

**Published:** 2024-01-25

**Authors:** Elisa Diral, Corrado Campochiaro, Alessandro Tomelleri, Gregorio M. Bergonzi, Umberto Pizzano, Maurilio Ponzoni, Lucia Bongiovanni, Paola Ronchi, Cristina Tresoldi, Silvia Rigamonti, Federico Scarfò, Gloria M. Latino, Emma Rinaldi, Massimo Bernardi, Lorenzo Dagna, Fabio Ciceri

**Affiliations:** ^1^ Unit of Hematology, IRCCS San Raffaele Scientific Institute, Milan, Italy; ^2^ Unit of Immunology, Rheumatology, Allergy and Rare Diseases, IRCCS San Raffaele Scientific Institute, Milan, Italy; ^3^ School of Medicine, Vita-Salute San Raffaele University, Milan, Italy; ^4^ Pathology Unit, IRCCS San Raffaele Scientific Institute, Milan, Italy; ^5^ Unit of Immunohaematology and Transfusion Medicine, IRCCS San Raffaele Scientific Institute, Milan, Italy

**Keywords:** myelodysplastic neoplasms (MDS), VEXAS syndrome, next generating sequencing, azacytidine, ruxolitinib, vacuoles

## Abstract

VEXAS syndrome is an acquired autoinflammatory disease characterized in most cases by cytopenias and macrocytic anemia. Dyshematopoiesis is a frequent finding in chronic inflammatory conditions and therefore, cytopenias are not easily classified in VEXAS patients. Here we report a series of 7 patients affected by VEXAS associated cytopenias, treated at our center. The use of NGS, together with morphological assays, integrated with the WHO 2022 criteria, allowed to identify three subsets of VEXAS associated cytopenias: ICUS (idiopathic cytopenia of uncertain significance), CCUS (clonal cytopenia of uncertain significance) at high risk of clonal evolution, and MDS. This approach could help to better understand the nature of VEXAS associated cytopenias and to guide the use of specific targeted treatments in order to achieve long lasting responses.

## Introduction

VEXAS (Vacuoles, enzyme E1, X-linked, autoinflammatory, somatic) syndrome is an autoinflammatory disease (1) caused by a somatic mutation in the UBA1 gene that leads to aberrant activation of the innate immune system and production of proinflammatory cytokines.

VEXAS syndrome is usually diagnosed in adult men with a median age between 65 and 75 years (2), and manifests with fever, skin vasculitis, pleuropulmonary disorders, polychondritis and ocular inflammation (3). Hematological involvement is very common and vacuolization of hematopoietic precursors is considered one of the hallmarks of the disease (4); indeed, the finding of at least two vacuoles in more than 10 per cent of neutrophil precursors is associated with high sensitivity and specificity for VEXAS syndrome diagnosis (5). Cytopenias are also frequent, with macrocytic anemia (1, 6) being the most common finding. In addition, patients with VEXAS syndrome present with myelodysplastic syndrome (MDS) in about 40% of cases (3). Studies have hypothesized that chronic inflammation leads to accelerated ageing of hematopoietic stem cells, favouring the onset of driver mutations usually belonging to the DTA triad (DNMT3A, TET2, ASXL1) (7). VEXAS associated MDS tend to show a low risk profile according to the Revised International Prognostic Scoring System (IPSS-R) (6, 8), even if evolution to acute myeloid leukemia has been reported (9). However, in VEXAS syndrome, cytopenias are not easy to classify, due to the frequent dyshematopoiesis found in bone marrow samples (typical of chronic inflammatory conditions) and to the presence of clonal hematopoiesis of indeterminate potential mutations (10). Typical marrow findings in VEXAS-associated MDS include hypercellular bone marrow, with cytoplasmic vacuolization and dysplasia in more than 10 per cent of erythroid and myeloid precursors; less frequently, vacuoles can be seen in plasma cells and megakaryocytes. Also, a variable degree of reticulin fibrosis, karyotype abnormalities, ring sideroblasts and acquired somatic mutations can be found. On the other hand, cytopenias without criteria for MDS also show cytoplasmic vacuolization of marrow precursors and bone marrow hypercellularity, with a minimal degree of dysplasia without blast excess and normal karyotype (6, 11). With regard to persistent and not otherwise explained cytopenias, the latest WHO classification of 2022 (12) has introduced two novel entities: clonal cytopenias of uncertain significance (CCUS), defined by the presence of clonal mutations with cytopenias in the absence of dysplasia (13), and idiopathic cytopenias of uncertain significance (ICUS), defined by cytopenias in the absence of clonal myeloid mutations (14). Actually, a precise classification of cytopenias in VEXAS syndrome is not available, due to the rarity of the disease and to the limited knowledge about pathogenic mechanisms (11). For this purpose, the new WHO 2022 classification could help in classifying VEXAS associated cytopenias, considering morphological, cytogenetic and molecular characteristics. This might help clinicians to better define the risk of clonal evolution of cytopenias and to choose proper treatments.

## Methods

In this study, we retrospectively analyzed bone marrow samples from 7 patients diagnosed with VEXAS syndrome at our centre between 2021 and 2022. Aspirates and trephine biopsies were performed at diagnosis and repeated during follow up in case of changes in blood counts. Morphological, cytogenetic, and molecular testing by next generation sequencing (NGS) were exploited. MDS, CCUS and ICUS were defined according to WHO 2022 criteria (12). All samples were collegially reviewed by a team composed by hematologists, morphologists and pathologists. Therapeutic decisions were performed by a multidisciplinary team of hematologists and rheumatologists.

## Results

The main features of the included patients are summarized in **Table 1**. All patients were men older than 60 years at diagnosis and presented with autoinflammatory symptoms and cytopenias. Two patients (28.5%) were diagnosed with low/intermediate risk MDS according to IPSS-R, without cytogenetic abnormalities or additional somatic mutations. Three patients (43%), who did not fulfil criteria for MDS and did not harbor somatic mutations or cytogenetic abnormalities, were diagnosed with ICUS. Two patients (28.5%), who did not fulfill criteria for MDS but with somatic mutations consistent with clonal hematopoiesis, were diagnosed with CCUS. All patients showed vacuolization of hematopoietic precursors, and all samples displayed a physiological karyotype.

**Table 1:** Classification of cytopenias in VEXAS syndrome.

	Timing of bone marrow evaluation	Classification WHO 2022	IPSS-R	NGS (VAF %) ┼	Karyotype	Clinical manifestations at diagnosis	Treatment
**PT1**	Diagnosis	CCUS		ZRSR2 (3.79%) DNMT3A (38%)	46, XY [23]	Cytopenia Orbital pseudotumor	GC + CSA
	Follow up	CCUS		ZRSR2 (3.47%) DNMT3A (37%) ASXL1 (2.12%)	46, XY [20]		GC + Ruxo
**PT2**	Diagnosis	CCUS		DNMT3A (39%)	46, XY [19]	Cytopenia Ear chondritis, orbital pseudotumor	GC + CSA
	Follow up	MDS-LB	Low	DNMT3A (40%)	46, XY [21]		GC + CSA + EPO
**PT3**	Diagnosis Follow up	ICUS ICUS		Negative Negative	46, XY [28] 46, XY [20]	Cytopenia Lung inflammation, cutaneous vasculitis	GC + tocilizumab * GC + Ruxo
**PT4**	Diagnosis	MDS-LB	Low	Negative	46, XY [24]	Cytopenia Ear and nose chondritis lung inflammation	GC + Ruxo *
	Follow up	MDS-LB	Intermediate	Negative	46, XY [20]		5-aza + Ruxo + GC
**PT5**	Diagnosis	MDS-LB	Very low	Negative	46, XY [18]	Cytopenia Ear chondritis, cutaneous vasculitis, lung inflammation	GC
	Follow up	MDS-LB	Low	Negative	46, XY [28]		GC + canakinumab + CSA + EPO
**PT6 §**	Diagnosis	ICUS		Negative	46, XY [22]	Cytopenia Ear chondritis, arthritis cutaneous vasculitis.	GC + tocilizumab
**PT7**	Diagnosis	ICUS		Negative	46, XY [20]	Cytopenia Ear chondritis	GC

ICUS, idiopathic cytopenia of undetermined significance; CCUS, clonal cytopenia of undetermined significance; MDS, myelodysplastic neoplasia; MDS-LB, myelodysplastic neoplasia with low blasts. GC, glucocorticoids; CSA, Cyclosporine A; Ruxo, ruxolitinib; EPO, erythropoietin; 5-aza, 5-azacytidine. ┼ Only oncogenic and potentially oncogenic variants are reported; § a second NGS testing performed during follow up on peripheral blood was negative. *Pretreated patients – here we indicate the last line of therapy before switching to novel therapies.

None of the three patients with ICUS had significant changes in blood counts during a median follow up of 11 months. At diagnosis, patient-3 (PT3) marrow evaluation showed mild hypoplasia with mild dyshematopoiesis; the patient underwent several lines of therapy and eventually achieved good clinical control with ruxolitinib. A second marrow evaluation performed before ruxolitinib initiation was still compatible with ICUS. PT6 had bone marrow hyperplasia without additional myeloid mutations. The patient achieved initial good disease control with low dose glucocorticoids and tocilizumab. However, about six months after diagnosis he had an aggressive inflammatory relapse with pulmonary, renal, and central nervous system involvement. The patient was refractory to high dose glucocorticoids and eventually died. A NGS assay ruled out clonal evolution at the time of death. PT7, currently lost to follow-up, was transfusion dependent due to macrocytic anemia; given his age and multiple co-morbidities, he was only treated with low-dose glucocorticoids.

Two patients were classified as CCUS. At diagnosis, PT2 had marrow hyperplasia with mild dyshematopoiesis and hemophagocytosis without criteria for MDS; the karyotype was normal and NGS testing showed an oncogenic mutation in DNMT3A. One year later, a bone marrow re-evaluation was performed due to a drop in hemoglobin levels despite controlled systemic inflammation, and it showed evolution to MDS with low blasts (MDS-LB) with no changes in karyotype and molecular mutational pattern. Hence, erythropoietin-stimulating agents (ESA) were added to the ongoing therapy with cyclosporine and low-dose glucocorticoids. PT1 had oncogenic mutations in DNMT3A and ZRSR2 at diagnosis and was treated with cyclosporine and low dose glucocorticoids. A marrow re-assessment performed six months after diagnosis for clinical worsening showed stable histological characteristics, with the appearance in NGS of a new oncogenic mutation in ASXL1. He was eventually switched to ruxolitinib.

The two patients who presented with MDS at VEXAS diagnosis had worsening cytopenias during disease course leading to an increased risk according to IPSS-R but without any signs of clonal evolution. PT4 initially presented with MDS-LB, normal karyotype and no myeloid additional mutations. He was initially treated with various combined therapies, including ESA, glucocorticoids, cyclosporine and canakinumab. One year after diagnosis, ruxolitinib treatment was started due to relapsing inflammatory events, increased transfusion requirement and progressive bone marrow failure. A good response in systemic inflammation was obtained, along with platelet count increase; however, due to persistent transfusion dependent anemia, 5-azacytidine was added. PT5 presented at diagnosis with MDS-LB and was initially treated with low-dose glucocorticoids monotherapy. Two years after diagnosis, he showed inflammatory signs and worsening anemia; a new bone marrow evaluation excluded clonal evolution, so a treatment with canakinumab, cyclosporine, low dose steroids and ESA was initiated with good clinical response.

## Discussion

**Figure 1:**
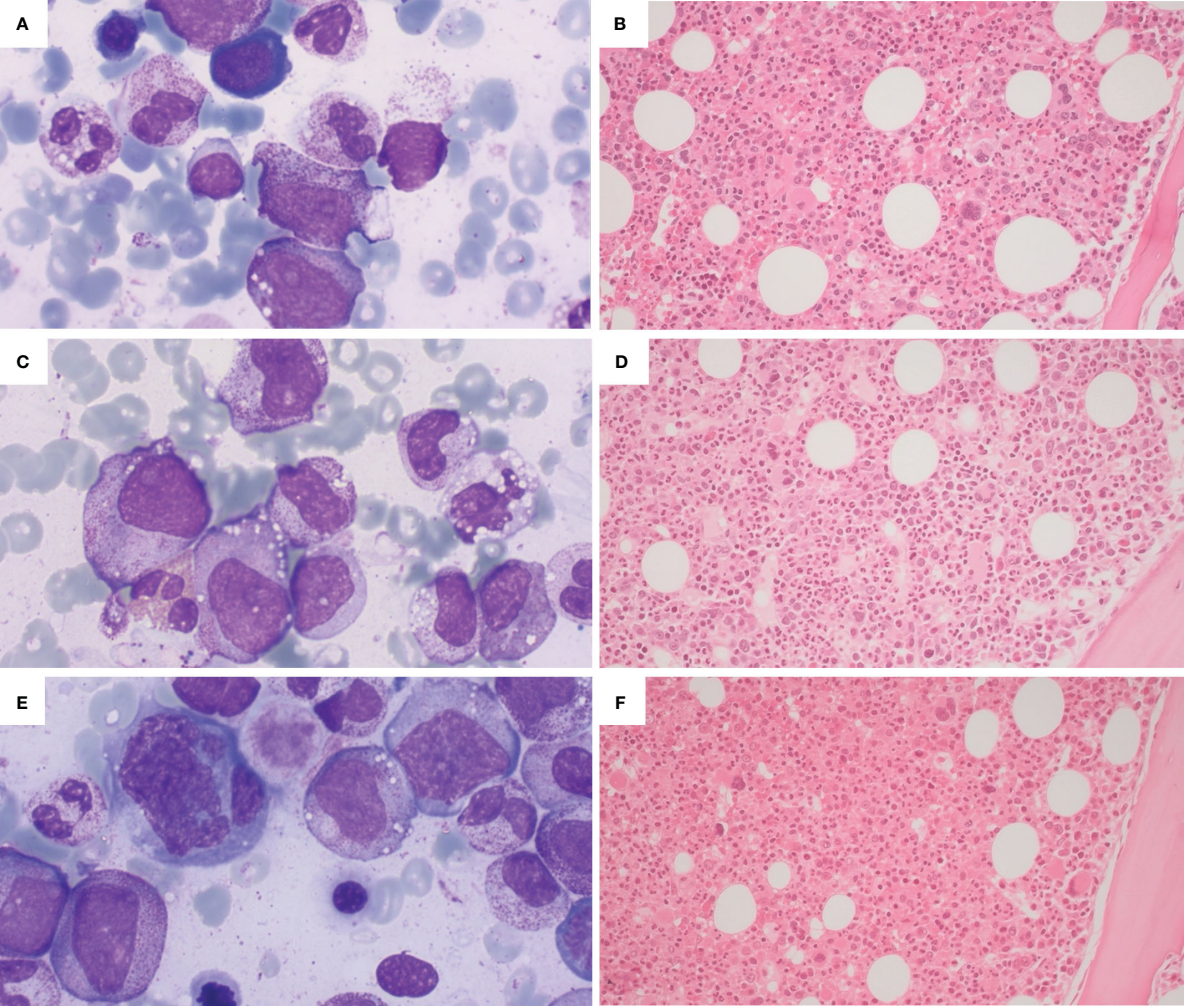
Illustrative bone marrow findings from VEXAS patients with cytopenias. **(A, B)** Cytological and histologic findings from a patient with ICUS (idiopathic cytopenia of undertermined significance): hypercellular bone marrow with maturating haemopoietic series, without dysplasia (**A** - Wright-Giemsa stain, 100x; **B** - H&E, 20x). **(C, D)** Cytological and histologic findings from a patient with CCUS (clonal cytopenia of undertermined significance), showing a hypercellulated bone marrow without morphological criteria for MDS (myelodysplastic neoplasia); presence of haemophagocytosis (**C** - Wright-Giemsa stain, 100x; **D** - H&E, 20x). **(E, F)** Cytological and histological findings from a patient with VEXAS and concomitant MDS, showing a markedly hypercellular bone marrow with trilinear dysplasia (**E** - Wright-Giemsa stain, 100x; H&E, 20x).

Our case series of bone marrow features in VEXAS-associated cytopenias shows high heterogeneity, as already reported in other works (11). Cytoplasmic vacuoles in myeloid and erythroid precursors, marrow hyper- or hypocellularity, varying degrees of dysplasia and hemophagocytosis are among these features. Given that a various grade of dyshematopoiesis is frequent in autoinflammatory disorders, it is not always easy to classify cytopenias in VEXAS syndrome (10). The use of molecular analysis by NGS, together with morphological assays, integrated with the WHO 2022 criteria, could help in this setting, by identifying mutations associated with clonal hematopoiesis and therefore patients at risk of clonal evolution. Moreover, the correct diagnosis of VEXAS associated cytopenias allows the use of specific targeted treatments, which might overcome the need for chronic moderate-to-high dose glucocorticoids and the lack of a prolonged response to conventional or biologic disease-modifying agents (15). Hematologic treatments are now used in VEXAS patients, such as ESA and 5-azacitidine; the latter has been successfully explored in a phase II trial in patients with VEXAS syndrome for the control of both inflammatory signs and cytopenias (16). For this reason, cooperation between hematologists, hematopathologists and rheumatologists is fundamental for both diagnosis and treatment optimization (15). Specifically, our experience classifies VEXAS associated cytopenias into three groups:

1) NGS-negative cytopenias (ICUS according to the WHO 2022 classification – **Figures 1A, B**), probably related to the inflammatory status (“VEXAS-associated cytopenia”);
2) “VEXAS-associated CCUS”, that is cytopenia associated with somatic mutations in myeloid genes but without morphologic criteria for MDS diagnosis, at high risk of clonal evolution (**Figures 1C, D**);
3) “VEXAS-associated MDS” (**Figures 1E, F**), that is cytopenia with marrow morphologic criteria for myelodysplastic neoplasia, with or without mutations in myeloid genes. In our case series, patients with VEXAS-associated MDS showed low risk of leukemic evolution but high risk of progressive bone marrow failure.

The prognostic value of cytopenias in VEXAS needs to be further investigated; however, the first case of evolution into acute leukemia has been recently described in a patient affected by VEXAS and MDS (9). Moreover, Ferrada and colleagues identified transfusion dependency among predictors of mortality in VEXAS syndrome (17). Our study has several limitations (i.e., small sample size, retrospective nature) and further studies are warranted to better characterize myeloid alterations in VEXAS patients and their relationship with the UBA1 clone. For example, incorporation of UBA1 to standard NGS diagnostic panels could help to determine the true prevalence of UBA1 mutations in patients with cytopenias.

## Conclusion

In our case series, we classified cytopenias in VEXAS syndrome according to the WHO 2022 classification. This approach could allow the implementation of personalized medicine in VEXAS syndrome, depending on the nature of the associated cytopenia and therefore improve patient outcomes. However, prospective studies are envisaged to deeply investigate the pathogenesis of the disease and effective treatments.

## Data availability statement

The original contributions presented in the study are included in the article/supplementary material. Further inquiries can be directed to the corresponding author.

## Ethics statement

Written informed consent was obtained from the individual(s) for the publication of any potentially identifiable images or data included in this article.

## Author contributions

ED: Conceptualization, Investigation, Methodology, Validation, Writing – original draft, Writing – review & editing. CC: Conceptualization, Methodology, Supervision, Writing – review & editing. AT: Conceptualization, Writing – review & editing. GB: Data curation, Writing – original draft. UP: Writing – original draft. MP: Resources, Writing – original draft, Writing – review & editing. LB: Resources, Writing – original draft, Writing – review & editing. PR: Resources, Writing – original draft, Writing – review & editing. CT: Resources, Writing – original draft, Writing – review & editing. SR: Resources, Writing – original draft, Writing – review & editing. FS: Resources, Writing – review & editing. GL: Writing – review & editing. ER: Writing – review & editing. MB: Writing – review & editing. LD: Writing – review & editing. FC: Writing – review & editing.
